# Ex Vivo Tools and Models in MASLD Research

**DOI:** 10.3390/cells13221827

**Published:** 2024-11-05

**Authors:** Rallia-Iliana Velliou, Eirini Giannousi, Christiana Ralliou, Eva Kassi, Antonios Chatzigeorgiou

**Affiliations:** 1 Department of Physiology, Medical School, National and Kapodistrian University of Athens, 75 Mikras Asias Str., 11527 Athens, Greece; rvelliou@med.uoa.gr (R.-I.V.); eirgiannousi@med.uoa.gr (E.G.); christrall@med.uoa.gr (C.R.); 2 Department of Biological Chemistry, Medical School, National and Kapodistrian University of Athens, 75 Mikras Asias Str., 11527 Athens, Greece; ekassi@med.uoa.gr

**Keywords:** ex vivo models, metabolic dysfunction-associated fatty liver disease (MASLD), liver-on-a-chip (LoC), organoids, ex vivo precision-cut liver slice (PCLS)

## Abstract

Metabolic dysfunction-associated fatty liver disease (MASLD) presents a growing global health challenge with limited therapeutic choices. This review delves into the array of ex vivo tools and models utilized in MASLD research, encompassing liver-on-a-chip (LoC) systems, organoid-derived tissue-like structures, and human precision-cut liver slice (PCLS) systems. Given the urgent need to comprehend MASLD pathophysiology and identify novel therapeutic targets, this paper aims to shed light on the pivotal role of advanced ex vivo models in enhancing disease understanding and facilitating the development of potential therapies. Despite challenges posed by the elusive disease mechanism, these innovative methodologies offer promise in reducing the utilization of in vivo models for MASLD research while accelerating drug discovery and biomarker identification, thereby addressing critical unmet clinical needs.

## 1. Introduction

Metabolic dysfunction–associated steatotic liver disease (MASLD), previously known as Non-Alcoholic Fatty Liver Disease (NAFLD), represents a significant and escalating global health challenge with an estimated prevalence of 25% worldwide affecting over 80% of individuals presenting with severe obesity [1]. MASLD is characterized by the accumulation of fat in the liver in the absence of significant alcohol consumption [2,3]. It encompasses a spectrum of liver conditions, ranging from simple steatosis to the more severe form of metabolic dysfunction-associated steatohepatitis (MASH) up to cirrhosis, which can give rise to hepatocellular carcinoma. The rising prevalence of MASLD is closely linked to the global increase in obesity, type 2 diabetes, and metabolic syndrome, making it a critical public health concern [4,5]. Despite its widespread impact, the pathophysiology of MASLD is complex and not fully understood, which complicates the development of effective therapeutic strategies.

A major issue in the management of MASLD has historically been the limited availability of therapeutic approaches [6]. Until recently, there were no FDA-approved medications specifically for the treatment of MASLD or NASH. Clinical management primarily focuses on lifestyle modifications, such as diet and exercise, to reduce liver fat and improve metabolic health. While these interventions can be beneficial, they are often difficult to maintain and may not be effective for all patients, especially for those in more advanced stages of the MASLD spectrum. However, the treatment landscape has begun to shift with the FDA’s recent approval of Rezdiffra (resmetirom) in March 2024 [7,8]. Despite this progress, the development of pharmacological treatments remains challenging due to the complex and multifactorial nature of the disease, highlighting the need for innovative research tools and models that can provide deeper insights into the disease mechanisms and facilitate the discovery and testing of new therapies [9]. Numerous approaches exist for studying MASLD, including animal models and non-invasive imaging techniques, which provide valuable insights into disease progression and the development of potential therapies [10,11]. Among these, ex vivo tools and models have emerged as crucial components in the research arsenal against MASLD. These models involve the study of liver tissue or cells outside their natural context, providing a well-controlled environment to explore the biological processes underlying MASLD [12]. An ex vivo model typically refers to a biological model derived from living tissue that is maintained outside the organism but still simulates in vivo conditions closely. Such models hold promise for improving MASLD diagnosis by identifying biomarkers for early detection and risk assessment, advancing therapeutic development by replicating disease complexity and enabling personalized treatments through patient-derived cells that account for individual genetic and metabolic factors. Ex vivo models, such as liver on a chip system, liver slices, and liver organoids, offer several advantages over traditional in vivo studies. They allow for detailed examination of cellular interactions, metabolic pathways, and drug responses in a system that closely mimics the human liver [13]. Success in establishing ex vivo models is determined by their ability to closely mimic human conditions while maintaining tissue viability and integrity, which enhances the predictability of drug absorption. Additionally, correlating system performance with in vivo outcomes is essential for validating model effectiveness [14]. By replicating the complex microenvironment of the liver, ex vivo models are instrumental in disease modeling, drug screening, biomarker discovery, and the development of personalized medicine approaches [15]. Key molecular mechanistic pathways involved in MASLD progression, such as the activation of hepatic stellate cells, the role of pro-inflammatory cytokines, and lipid accumulation in hepatocytes, can be systematically explored using ex vivo models [15].

**Figure 1:**
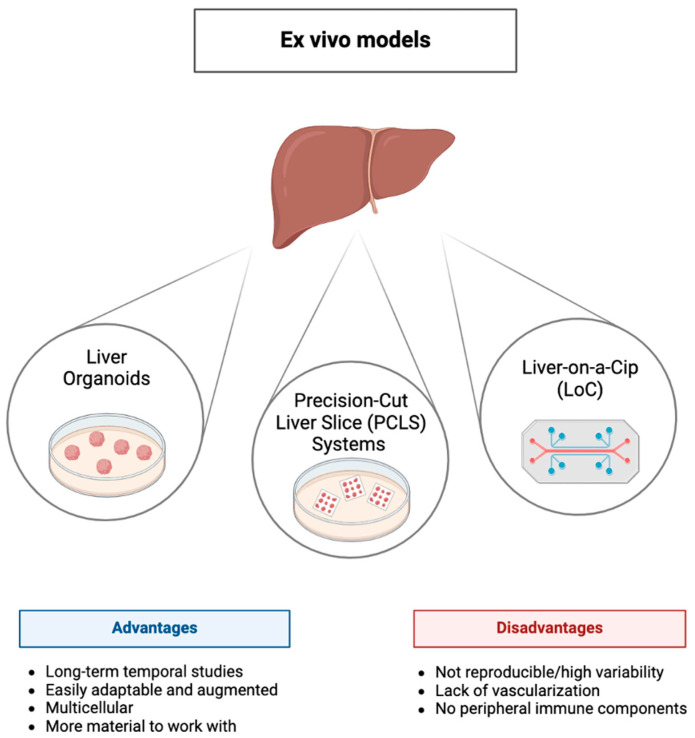
Overview of ex vivo liver models: The diagram highlights three common ex vivo liver models: Liver Organoids, Precision-Cut Liver Slice (PCLS) Systems, and Liver-on-a-Chip (LoC). Each model has distinct advantages and disadvantages. Liver organoids are multicellular and can be easily adapted or augmented, allowing long-term temporal studies with more available material. PCLS systems preserve tissue architecture but may suffer from variability. LoC technology provides a more physiologically relevant microenvironment, but limitations include lack of vascularization and absence of peripheral immune components. These models collectively contribute to advancing liver research, with varying suitability based on experimental needs.

As the landscape of MASLD research expands, there is an urgent need to integrate findings from ex vivo studies into clinical applications, paving the way for more effective and personalized treatments. To that end, we aim to explore the applications and challenges of ex vivo models and highlight how these models contribute to our knowledge of MASLD pathophysiology, support the development of new therapeutic strategies, facilitate the identification of biomarkers, and enable personalized medicine approaches. Additionally, we will address the technical challenges and translational barriers associated with the use of ex vivo models and discuss potential solutions to enhance their utility and relevance in clinical settings (Figure 1). By providing a comprehensive overview of the current state and future prospects of ex vivo models in MASLD research, this review seeks to underscore their importance and inspire further advancements in this vital area of study.

## 2. Liver-on-a-Chip (LoC) Systems

Traditional cell culture systems and in vivo models have long been employed to investigate the underlying mechanisms of MASLD pathogenesis. However, these approaches fall short of accurately reproducing the entire spectrum of complex liver processes and unique three-dimensional (3D) microenvironment, internal vascular system, and cell-cell interactions involved in MASLD pathophysiology [16]. As a result, there is a growing demand for models capable of reproducing the complexity of liver disease physiology and hepatic tissue microenvironment. This need enabled a rapid advancement in sophisticated microphysiological systems, where human and murine cell lines are combined with bioscaffolds to generate functional 3D tissue microenvironments that are suitable to investigate the progression of liver disease, aid in drug discovery, and facilitate toxicity testing [13,17].

### 2.1. Design and Methodology of LoC Systems

Liver-on-a-chip (LoC) systems are advanced 3D in vitro microphysiological models designed to replicate the complex conditions of hepatic tissue ex vivo. These systems emulate the dynamic physicochemical interactions of the hepatic microenvironment, including the architecture of liver lobules with hepatic cords, sinusoid-like structures, and polarized hepatocytes [17]. This provides a valuable platform for studying the pathophysiology of liver diseases, drug screening, and development, as well as for investigating multi-organ interactions. MASLD and various other liver injuries involve multiple organ systems and are characterized by significant increases in circulating inflammatory biomarkers [18]. The experimental set-up of LoCs facilitates the collection of culture medium circulating through the perfusion system for further analyses.

The preparation of the LoC system involves five key steps [19]. First, patterns are produced using computer-aided design (CAD)-based software. Second, an expert template is created on the chip using methods involving mask lithography, maskless lithography, femtosecond laser etching, or laser welding [20]. The third step focuses on constructing the LoC, utilizing methods such as soft lithography, 3D bioprinting, and micropatterning, with soft lithography being the most prevalent. This technique involves replicating patterns from micromachined templates using elastic polymers like PDMS, which can be sealed in various configurations to create multilevel chips [21]. Meanwhile, 3D bioprinting combines biomaterials with printing technology to form physiologically relevant 3D tissue structures, though its resolution can be limiting [22]. The fourth step is plasma bonding of the chip materials, followed by equipment testing in the fifth step. Finally, the last step includes evaluating and testing the LoC equipment along with surface treatment for cell culture materials [23].

Over the years, LoC devices have successfully mimicked several hallmarks of MASLD. For instance, treatment with free fatty acids and glucose has been shown to induce fat accumulation in hepatocytes within the LoC microenvironment. Various compounds, including obeticholic acid, elafibranor, pioglitazone, and metformin, have been utilized to study their therapeutic effects in these in vitro settings.

### 2.2. Applications in MASLD Pathophysiology

Recent advancements in LoC technology have provided powerful tools for studying MASLD pathophysiology, enabling more precise modeling of liver functions. A notable development in LoC technology is the Metabolic Patterning on a Chip (MPOC) platform developed by Kang et al. [24]. This device is capable of mimicking the metabolic characteristics of liver tissue [24]. The MPOC platform includes a microfluidic gradient generator and a microfluidic tissue culture chamber with two orthogonal axes. By controlling concentration gradients, it recapitulates the hepatocytes’ zonation and functions, including carbohydrate, glycose, and nitrogen and xenobiotic metabolism [24]. Specifically, either primary rat or primary human hepatocytes were cultured in the MPOC system for 24 h, and the culture medium was supplemented with glucagon, insulin, and/or 3-methylcholanthrene to induce a pattern of metabolic functions [24]. The proposed MPOC device successfully generated stable gradients of different metabolic modulators such as hormones and inducers and created controlled metabolic patterns using actively enforced concentration gradients in a continuous microfluidic liver tissue model of both rat and human species, along with direct sectional characterization of different metabolic activities. Similarly, Bulutoglu et al. adapted the MPOC platform using rat primary hepatocytes [25]. Specifically, they created an oxygen gradient and exposed the hepatocytes to different FFA concentrations to examine the role of oxygen on lipid accumulation and MASLD progression. Their study demonstrated that oxygen deprivation has a more pronounced effect on lipid accumulation at lower free fatty acid (FFA) levels, but this effect diminishes as FFA availability increases and lipid accumulation saturates [25]. Long-term hepatic cell culture is an important tool for studying drug metabolism in vitro [23]. The use of a microfluidics device or perfusion-based device can collect and recirculate hepatic biomarkers, which was impossible to achieve using conventional methods. LOC platforms can help sustain the phenotype of hepatocytes and liver-specific functions in long-term culture [26]. Several studies have utilized LoC devices to model MASLD ex vivo by treating specific hepatic cell types with FFA. For example, Kostrzewski et al. co-cultured primary human hepatocytes, Kupffer cells, and hepatic stellate cells (HSCs) in a perfused 3D microphysiological system (MPS) [27,28]. By culturing the microtissues in a medium containing FFAs for at least two weeks, they induced a MASLD phenotype characterized by hepatic fat accumulation and an inflammatory and fibrotic environment [27]. This model demonstrated that obeticholic acid alleviates the MASH phenotype. Additionally, they identified that the patatin-like phospholipase domain containing 3 (PNPLA3) I148M mutation in HSCs aggravates the disease. Co-cultures with mutant I148M PNPLA3 HSCs displayed enhanced pro-inflammatory responses but did not affect the fibrotic phenotype. This specific genetic signature could be used as a risk factor marker for the disease progression. This LoC setup enables co-cultures of specific cell types in a highly functional state for 2 weeks and allows the investigation of numerous molecular pathways [27].

In another advancement, Li et al. created a novel vascularized human liver acinus MPS (vLAMPS) containing primary human hepatocytes, liver sinusoidal endothelial cells (LSECs), and human cell lines for stellate and Kupffer cells [29]. This device forms a vascular channel that mimics the liver acinus, allowing communication between channels and recreating liver oxygen zonation. By controlling the flow rates of media through hepatic and vascular channels, they replicated the oxygen zonation present in the liver acinus, enabling investigations into its role in physiology, toxicology, and disease progression [29].

Furthermore, in another LoC device, four types of human primary liver cells—hepatocytes, Kupffer cells, LSECs, and HSCs—were co-cultured under microfluidic dynamics [30]. Exposure to a culture medium with FFAs, oleic acid (OA), palmitic acid (PA), and/or lipopolysaccharides (LPS) developed a MASH phenotype, including hepatic fat accumulation, hepatocyte ballooning, HSC activation, and elevated inflammatory and profibrotic marker expression. Treatment with elafibranor, a candidate drug against MASLD, suppressed the MASLD phenotype by decreasing lipid accumulation, ballooning, and the expression of inflammatory and fibrotic genes, while also inhibiting HSC activation [30]. In another study, Lasli et al. developed an in vitro system of MASLD by co-culturing human hepatocellular carcinoma (HepG2) and umbilical vein endothelial cells (HUVECs) into spheroids which were transferred onto a chip system with hexagonal microwells [31]. Supplementing the culture media with FFAs led to the development of steatosis in the spheroids. This steatosis-on-a-chip model, which combines the co-culture of hepatocytes and HUVECs, enhances the complexity, resulting in a more biologically relevant steatosis model capable of monitoring hepatic functionality and screening multiple drugs [31]. Another study utilized human induced pluripotent stem cells (hiPSCs) in a liver organoid-on-a-chip system [32]. Prior to the formation of the system, hiPSCs were induced into hepatic progenitors and were later differentiated by adding several growth and differentiation factors. Following the liver-organoids-on-chip formation, the culture medium was supplemented with FFAs for up to 7 days [32]. Once exposed to FFAs, liver-organoids-on-chip showed lipid droplet formation, triglyceride accumulation, and increased expression of lipid metabolic-related markers. Besides the aforementioned key traits of MASLD, organoids displayed an increase in the production of reactive oxygen species (ROS) and upregulation at the expression of proinflammatory and fibrotic genes. Wang et al. provided a novel platform with the typical pathological MASLD characteristics that enables in situ hepatic differentiation, formation, and long-term 3D culture of functional liver organoids and gives the opportunity to study disease mechanisms and drug compounds [32].

Du et al. designed a liver lobule chip that mimics dual blood supply via the scaffold-designed hepatic portal vein and hepatic artery [33]. Specifically, they incorporated human liver cell lines into the system, including HepaRG progenitor cells, LX2 (HSCs) cells, and Human Hepatic Sinusoidal Endothelial Cells (HHSECS). To simulate liver zonation, the medium was injected through inlets resembling the hepatic artery and portal vein and drained via outlets mimicking the central vein. MASLD induction was achieved by adding a lipogenic medium containing glucose and FFAs for up to 7 days. Following the treatment, the amount of lipid droplets within the zone 1-like region was higher than that of the zone 2-like region; however, following MASLD progression, the lipid zonation gradually disappeared [33]. This study is the first to demonstrate the changing lipid zonation in a liver lobule chip at the early stages of MASLD and provides a more accurate platform for studying pathophysiological mechanisms and drug screening [33].

Lastly, Slaughter et al. developed a complex microfluidic adipose-liver human-on-a-chip (HoaC) model composed of both human hepatocytes and adipocytes [34]. This system contains primary human hepatocytes and primary human cardiac preadipocytes differentiated into mature adipocytes. Hepatocytes and adipocytes were co-cultured in a 2-chamber system for 2 weeks, supplied with different kinds of blood-mimetic medium, including a diabetic medium containing elevated glucose and insulin concentration, a proinflammatory medium supplemented with tumor necrosis factor-alpha (TNFa), and an obese medium with high concentrations of FFAs [34]. Higher lipid accumulation and steatosis were observed when the proinflammatory and obese media were combined in the 2-chamber system. Although in the hepatocyte’s monoculture, TNFa treatment did not increase steatosis, the presence of steatosis was prominent in the 2-chamber system. This is consistent with the fact that in vivo TNFa is mostly secreted by macrophages that infiltrate adipose tissue [34,35,36]. Moreover, the proinflammatory and proinflammatory/obese medium impaired CYP3A4 activity in hepatocytes, a result that aligned well with the CYP3A4 activity found in MASH patients and in in vitro MASH models. Lastly, this platform was utilized to examine the effect of metformin, a drug for T2D patients [37]. Metformin significantly decreased steatosis only at higher concentrations than physiological doses; however, it was accompanied by hepatocyte toxicity and cell death. Thus, this novel HoaC provides a valuable interorgan platform to study hepatocyte-adipocyte interactions and evaluate drug responses [34].

### 2.3. Limitations and Challenges of LoC in MASLD Modeling

While LoC systems hold great promise in modeling MASLD, they come with several limitations. The high complexity and cost of LoC technology limit its widespread application, making it an expensive and technically challenging endeavor [12]. Additionally, current LoC systems often provide only end-point results, missing critical physiological processes and dynamic changes during disease progression [38,39]. This limitation hampers the ability to capture the full spectrum of disease mechanisms and temporal evolution. Furthermore, LoC devices cannot replicate inter-organ communication or the complex immune microenvironment, limiting the study of pathogenetic mechanisms and drug efficacy in a broad context of multi-organ physiology. Inter-organ interactions are crucial for understanding systemic responses and crosstalk between different tissues, which are particularly important in MASLD in the context of metabolic dysfunction and inflammation.

Future advancements are expected to produce multi-organs-on-chips, such as liver-kidney, liver-intestine, and liver-immune chips, ultimately aiming for a human-on-a-chip. Integrating multiple biosensors and employing high-tech detection technologies can enhance readouts and throughput, making LoC systems more efficient and comprehensive. These advancements could significantly improve the physiological relevance and translational potential of LoC models in MASLD research. Furthermore, the incorporation of advanced imaging techniques, real-time monitoring capabilities, and high-throughput screening methods will enhance the utility of LoC platforms in studying MASLD and testing therapeutic interventions.

## 3. Precision-Cut Liver Slice (PCLS) Systems

Precision cut liver slice (PCLS) is a novel ex vivo model for studying liver physiology, disease mechanisms, and pharmacological responses. PCLS offers the unique ability to obtain tissue sections while preserving the hepatic tissue organization, architecture, and multicellular composition of the liver, including hepatocytes, Kupffer cells, HSCs, and endothelial cells, providing a highly relevant system for investigating liver-specific processes. PCLS was first introduced in 1985 by Smith et al. as a 3D in vitro model of the liver, offering potential as it holds all the necessary requirements to mimic many aspects of the in vivo liver structures [40,41]. The preparation of PCLSs involves several key steps to ensure the preservation of liver architecture and cell viability. Initially, a liver is rapidly excised and perfused with a cold preservation solution to minimize ischemic damage. The liver is then embedded in low melting point agarose to provide structural support. Using a precision slicer, typically a vibratome, thin slices (150–250 µm) are cut from the agarose-embedded liver. These slices are then placed in a culture medium under controlled conditions, allowing for prolonged maintenance of liver functionality [40,41]. The advantages of this model were instantly recognized, particularly because hepatocyte cultures at the time lacked the heterogeneity of liver cells and, therefore, could not adequately replicate the in vivo liver [40]. Initially, PCLS cultures were utilized for metabolic studies and toxicity testing, but from 2005 onwards, the focus of PCLS experiments shifted from toxicity testing to investigating chronic liver diseases, such as fibrosis [42]. Human PCLSs are usually obtained from partial hepatectomy or diseased livers from patients with severe fibrosis and cirrhosis. Moreover, resected liver tissue can be obtained from patients with primary or metastatic liver cancer [43,44]. PLSCs provide the advantage of preserving the complex 3D liver tissue architecture (native extracellular matrix components), including cell–cell and cell–matrix interactions as well as heterotypic interactions found in vivo. They also preserve the viability of hepatocytes, Kupffer cells, endothelial cells, and HSCs for up to 5 days and up to 15 days under optimal conditions. PCLSs are particularly well-suited for modeling liver diseases in general, including MASLD as well as alcoholic liver disease (ALD), fibrosis, and hepatocellular carcinoma (HCC). By exposing liver slices to specific disease-relevant stimuli (e.g., FFAs, ethanol, toxins), researchers can induce pathological changes that mimic those observed in vivo. By using culture media containing glucose, fructose, insulin, and/or FFAs, PCLSs can exhibit phenotypic characteristics of MASLD, namely hepatic steatosis, lipid deposition, and lipotoxicity [43]. For instance, PCLSs can be treated with a mixture of FFAs (oleic and palmitic acid) to induce steatosis, inflammation, and subsequent fibrosis. This allows for the investigation of lipid metabolism, inflammatory pathways, and fibrotic responses, providing insights into disease progression and potential therapeutic targets. Thus, PCLSs, either from humans or mice, can be a valuable ex vivo model to study disease mechanisms and test drug compounds. To that end, several studies have utilized PCLSs obtained from experimental animals to explore molecular and multicellular mechanisms of MASLD and examine the effect of potential therapeutic compounds [45,46,47].

### 3.1. Applications of Rodent PCLSs in MASLD Pathophysiology

Current in vitro MASLD models fail to represent pathophysiological mechanisms that drive the disease and do not reflect the crucial cellular interactions that occur during the MASLD progression. To overcome these limitations, several studies utilized PCLSs obtained from rodents’ livers. Prins et al. generated an ex vivo model of steatosis by culturing PCLSs obtained from Wistar rats’ livers in various combinations of culture media containing glucose, fructose, insulin, and/or PA for 24 and 48 h [48]. After 24 h, lipid droplet accumulation was prominent, while key genes of de novo lipogenesis, namely acetyl-CoA carboxylase 1 (ACC1) and 2 (ACC2), and sterol-responsive element binding protein 1c (SREBP-1c) were upregulated. Additionally, PCLSs cultured for 24 and 48 h in the aforementioned additives displayed reduced expression of carnitine palmitoyltransferase 1 (CPT1), a key transporter of long-chain fatty acids to the mitochondrial matrix, indicating impaired fatty acid transport and dysregulated mitochondrial β-oxidation. In this PCLS model, steatosis was indeed observed but did not induce an inflammatory or fibrotic response; hence, it could be a pivotal model to specifically investigate anti-steatotic drugs [48]. Based on the findings of the aforementioned PCLS model, Prins et al. cultured mouse PCLSs for 24 and 48 h in varying concentrations of fructose, glucose, insulin, PA, OA, and/or butyrate addition [49]. Studies have shown that low levels of butyrate are associated with increased hepatic inflammation and steatosis. Treatment with butyrate in the steatotic PCLSs did not alter triglyceride accumulation but reduced fatty acid oxidation-related and profibrotic genes [49]. These results imply that butyrate does not directly affect the pathogenesis of the disease, and its beneficial role may be affected by inter-organ communication [49]. In another study, Gore et al. induced MASH in C57BL/6 mice following a choline-deficient l-amino acid-defined (CDAA) and amylin (AMLN) diet for 12 and 26 weeks, respectively [50]. PCLSs prepared from these mice were cultured for 48 h. These PCLSs displayed enhanced gene expression of fibrosis markers, namely collagen type I alpha 1 chain (COL1A1), serpin family H member 1 (SERPINH1), and Fibronectin 1 (FN1). Inflammation was also present in PCLSs as shown by the increased gene expression of interleukin 1 beta (IL1B), interleukin 6 (IL6), and TNFa, known inflammatory markers, while decreased expression of acyl-CoA oxidase (ACOX), carnitine palmitoyltransferase 2 (CPT2A), and peroxisome proliferator-activated receptor alpha (PPARA) indicated reduced lipid metabolism. In order to further enhance the replicability of MASLD pathology ex vivo, PCLSs were also cultured for 48 h with modulators of inflammation (LPS) and fibrosis (TGFβ1), which have been linked to MASLD in patients, and elafibranor, a PPARα/δ agonist and potential treatment for MASLD [50]. LPS and TGFβ1 treatment further inducted fibrosis and inflammation, as shown by the increased gene expression of the relative markers and negatively affected the lipid catabolism. On the other hand, elafibranor did not appear to improve the state of fibrosis and inflammation but triggered the modulation of lipid and carbohydrate metabolism due to PPARα/δ activation. This study highlights the potential of PCLSs as a promising and useful tool for preclinical evaluation of potential therapeutic compounds [50].

As previous studies have shown, the role of hyperammonemia is evident in MASLD. To that end, De Chiara et al. utilized rat PCLSs cultured with FFAs and/or ammonia for 24 and 48 h to induce steatosis and fibrosis [51]. The gene expression of fibrotic markers such as a-SMA and COL1A1 was indeed increased following lipid and/or ammonia treatment, while also collagen deposition in the PCLSs was evident, as observed histologically using the PicroSirius Red staining [51]. The addition of the ammonia scavenger ornithine phenylacetate (OP) for lowering ammonia levels resulted in a significant depletion of fibrosis and, at the same time, reduced fibrotic markers expression. This study determined that ammonia has a key role in fibrogenesis, while OP can prevent the progression of hepatic fibrosis [51].

### 3.2. Applications of Human-Derived PCLSs in MASLD Pathophysiology

In addition to liver slices obtained from experimental models that investigate pathophysiological mechanisms and potential therapeutic compounds in MASLD, several studies have explored the use of human PCLSs. These studies aim to understand the mechanisms underlying MASLD development and progression directly in human specimens, thus holding the potential for characterizing and evaluating novel therapeutic targets. To that end, Shepherd et al. investigated the role of the hepatic amine oxidase enzyme vascular adhesion protein-1 (VAP-1) in steatosis, metabolic disturbance, and inflammation [52]. Specifically, human PCLSs obtained from healthy livers were exposed to OA for 24 h and cultured with recombinant VAP-1 or VAP-1 substrates, either endogenous (methylamine) or exogenous (benzylamine). Specifically, PCLSs treated with recombinant VAP-1 and its substrate methylamine exhibited elevated lipid accumulation, while the secretion of triglycerides into the culture supernatant from the slices was reduced. This indicates that VAP-1 activity promotes lipid uptake and inhibits the export of triglycerides from hepatocytes. Treatment with bromoethylamine, which inhibits VAP-1 activity, resulted in the reduction of steatosis, whilst the addition of methylamine to activate VAP-1 induced an inflammatory response [52]. VAP-1 has been proven to contribute to hepatic inflammation and fibrosis during MASH. This study shows an additional role of VAP-1 in hepatic steatosis, suggesting that inhibition of its oxidase capacity could pose a useful therapeutic target [52]. In a similar attempt, Bhattacharya et al. employed computational drug repurposing combined with direct testing on human PCLSs from fibrotic livers to identify a novel therapeutic candidate [53]. They tested the effect of AZD3355, a GABA-B receptor agonist known for treating esophageal reflux. Treatment of PCLSs with this compound for 24 h downregulated the expression of fibrotic genes (COL1A1, αSMA) and the inflammatory marker TNFa. This study highlights the potential of using human PCLSs to evaluate therapeutic compounds emerging from drug repurposing [53].

Mabire et al. examined whether targeting Mucosal-Associated Invariant T (MAIT) cells could function protectively against liver fibrosis [54]. In human PCLSs obtained from patients with end-stage fibrosis, MAIT cells were found to be localized near activated hepatic stellate cells [54]. Treatment with an inhibitor of MAIT activation, the non-agonist synthetic folate derivative Acetyl-6-formylpterin (Ac-6-FP), for 48 h significantly reduced the expression of inflammatory and fibrogenic genes (CCL2, COL1A1, COL1A2, IL1B, TGFB1, and TNFa) and decreased the number of α-SMA+ cells. This response demonstrates that MAIT cell inhibition promotes fibrosis regression in human liver tissues and, therefore, could serve as a therapeutic target [54]. Simon et al. conducted a comprehensive transcriptomic profiling of induced steatosis in both human and mouse PCLSs [55]. Both human and mouse PCLSs were cultured with incremental supplementation of sugars (glucose and fructose), insulin, and FFAs (PA and OA) for 24 and 48 h. In human PCLSs, there was significant variability across different liver donors, whereas mouse PCLSs showed less variability and were more influenced by these experimental conditions. RNA-Seq analysis revealed conserved genes that were consistently up- or down-regulated based on culture time, as well as genes and pathways strongly regulated by steatosis induction. Notably, some genes exhibited opposite expression patterns in humans versus mice, highlighting species differences in response to steatosis. This study provides valuable data on the ex vivo use of PCLSs in the context of steatosis [55]. Mei Li et al. cultured human PCLSs in a medium enriched with high sugar, high insulin, and high fatty acids for up to 96 h to induce MASLD [47]. The PCLSs displayed macrovesicular steatosis along with accumulation of intracellular fat, and triglycerides. Moreover, treated PCLSs demonstrated increased pro-inflammatory and profibrotic gene expression. Focusing on the fatty acid uptake and metabolism in the treated PCLSs, fatty acid transport was upregulated at the first 24 h, but after 72 h, it started to decline due to cellular stress or damage [47]. RNASeq analysis showed that both TNFa and TGFβ signaling pathways contribute to the development of fibrosis and inflammation, confirming that decreased lipogenesis may have a role in liver damage and MASLD progression [47].

### 3.3. Limitations and Challenges of PCLSs in MASLD Modeling

Despite the numerous capabilities PCLSs provide for modeling MASLD, their application does pose a few limitations. Firstly, the lack of interaction with other organs involved in MASLD, such as adipose tissue, along with circulating immune cells and adipokines, limits the extent to which these models can accurately replicate human physiology. Moreover, the estimated lifetime of PCLSs in most setups is up to 5 days on average, which restricts the ability to observe long-term disease progression and responses to treatment [38,43]. Recent studies have shown that incorporating liver slices into microfluidic devices allows for the detection of unstable drug metabolites through high-performance liquid chromatography and extends tissue viability to several days in culture [56]. The primary benefit of using liver slices lies in their ability to maintain the liver’s heterotypic interactions and architecture, facilitating multiple experiments on a single organism and significantly reducing variability [57]. However, as previously mentioned, liver slices can only be sustained in culture for a limited number of days. Lastly, there are limitations regarding access to freshly resected human tissue, while at the same time samples from cirrhotic and HCC patients having undergone several therapeutic schemes prior to surgery, can compromise the integrity and properties of the obtained tissue leading to low cell density and rapid loss of cell function [58].

## 4. Organoids

The term “organoid” encompasses a range of 3D culture systems derived from tissue-resident stem/progenitor cells, embryonic stem cells (ESCs), or induced pluripotent stem cells (iPSCs) that replicate the morphological and functional characteristics of their tissue of origin [59,60]. These models significantly bridge the gap between traditional 2D cultures and in vivo mouse or human models, providing enhanced physiological relevance [61]. Cells cultured in 3D replicate the architectural and functional properties of in vivo tissues better due to the establishment of cell–cell and cell–extracellular matrix (ECM) interactions in all three dimensions. Furthermore, 3D culture techniques better emulate the in vivo environment, where cells are exposed to concentration gradients of signaling molecules, nutrients, and waste products [62]. Organoids can be generated from both healthy and diseased tissues, expanded long-term while maintaining genetic stability, and cryopreserved to create biobanks [63]. Recent advancements have enabled the development of 3D cultures of single or multiple cell types that successfully replicate functional in vivo-like liver structures [64]. Liver organoids, in particular, have shown immense potential for modeling patient-specific diseases and establishing personalized therapeutic approaches [65]. They have also been used in testing drug efficacy and toxicity. This overview highlights the cell sources for generating liver organoids, the benefits, and limitations of each cell type, as well as the application of the organoids in modeling liver diseases and validating drug safety and effectiveness.

### 4.1. Sources of Liver Organoids

Liver organoids can be derived from various sources, including stem cells, cancer cell lines, and primary cells, each offering unique benefits and applications [65]. Progenitor cells from both adult and fetal liver tissue can be induced to form organoids of the hepatocyte or cholangiocyte lineage upon incubation with specific combinations of growth factors. Additionally, progenitor cells isolated from bile can be cultured to grow into cholangiocyte organoids. Pluripotent stem cells (PSCs), whether of embryonic or somatic origin, require a three-stage differentiation protocol to produce hepatoblast-like cells, which are then embedded in an ECM to facilitate 3D growth and organoid formation [59]. Moreover, liver organoids derived from cancer cell lines, such as hepatocellular carcinoma or cholangiocarcinoma, are valuable for modeling liver cancer, enabling drug discovery and testing [60]. Lastly, primary human hepatocytes isolated from liver tissue can closely mimic in vivo liver function, making them useful for patient-specific studies and personalized hepatotoxicity models.

#### 4.1.1. Stem Cell-Derived Liver Organoids

Stem cell-derived liver organoids can be generated from four types of stem cells, including progenitor cells from adult and fetal liver tissue, human embryonic stem cells, and iPSCs [66]. Adult liver tissue-derived organoids are generated from progenitor cells that reside in the liver’s ductal and parenchymal compartments. These cells can differentiate into both hepatocytes and cholangiocytes [67]. The process begins with the isolation of these cells from human liver tissue, followed by their incubation in a culture medium enriched with growth factors such as hepatocyte growth factor (HGF), fibroblast growth factors (FGF), and inhibition of TGF-β signaling, through small molecule inhibitors, such as A8301 to allow for long-term expansion [68]. However, individual molecules are added depending on the cell type: forskolin for cholangiocyte organoids (chol-org) and GSK3B- and ROCK1 inhibitors for hepatocyte organoids (hep-orgs). Potentiation of Wnt signaling by R-spondin 1 is required for human and mouse chol-org, as well as human hep-org expansion, but not for mouse hep-org expansion [69]. These factors promote the proliferation and differentiation of progenitor cells, leading to the formation of three-dimensional organoid structures that mimic the cellular organization and functionality of the liver. Extracellular matrix hydrogels such as Matrigel or Cultrex Basement Membrane Extract provide structural support to the growing organoids and enable their 3D suspended growth [70].

Fetal liver tissue-derived organoids are generated from progenitor cells present in the developing liver. These cells are highly proliferative and possess a greater capacity for differentiation compared to their adult counterparts, reflecting the dynamic nature of fetal liver development [66]. The process of generating fetal liver organoids involves isolating these progenitor cells from fetal liver tissue and culturing them in a medium supplemented with growth factors that support their growth and differentiation into hepatocytes and cholangiocytes. Factors such as bone morphogenetic proteins (BMPs), HGF, and FGF play crucial roles in this process, guiding the cells through stages of liver development akin to those occurring in vivo [68]. The fetal progenitor cells have to also be treated with a medium supplemented with a Notch inhibitor, DAPT, to induce bile paucity [71]. Hep-orgs proliferate much slower and double every 5–7 days if derived from fetal livers or are passaged 1–2 times every 50–75 days if derived from adult livers [72]. Adult liver organoids retain characteristics specific to mature liver cells, making them ideal for studying diseases that manifest later in life and for personalized medicine applications. In contrast, fetal liver organoids provide a window into early liver development and are useful for modeling congenital diseases and studying developmental biology.

Fully functional organoids have been successfully derived from iPSCs. There are several protocols for the stepwise differentiation of iPSCs into hepatocyte-like cells (HLCs), generally consisting of three main stages [66]. The process typically begins with the induction and commitment of iPSCs to the endodermal lineage using an Activin A-rich media. This stage results in the up-regulation of posterior foregut (PFG)-specific markers, such as PDX1, HNF4A, and CDX2, and the downregulation of pluripotency markers, such as OCT4 and SOX2. During the second step, these endodermal cells further differentiate into hepatoblasts. This is achieved by treating the cells with factors such as BMP4, BMP7, and FGF7, which are known to drive hepatoblast differentiation in early development. The hepatoblasts express markers such as HNF4A, SOX9, EPCAM, TBX3, PROX1, AFP, and CK18 [64]. The final stage involves the maturation of hepatoblasts into HLCs by their incubation in a culture medium enriched with growth factors such as HGF, FGF, Wnt signaling molecules, and inhibition of TGF-β signaling. This medium also includes other factors like dexamethasone, CHIR99021, and A83-01 to support the maturation process. Unlike earlier methods, this protocol enables high-throughput generation of organoids that are consistent in shape, size, and structure and can be performed in a matrix-free environment [64]. Besides hepatocytes, iPSCs can also be differentiated into cholangiocytes, stellate-like, and Kupffer-like cells [73]. The pioneering work to create 3D liver tissue that mimicked embryonic liver features was conducted by Takebe and colleagues, who established embryonic liver bud cultures by co-culturing human iPSC-derived hepatocytes with human mesenchymal stem cells (MSCs) and human umbilical vein endothelial cells (HUVECs) [74]. One significant challenge in organoid models is the co-culture of multiple cell types with different media requirements. Recently, hepatobiliary structures containing both hepatocyte and cholangiocyte cells have been derived from human iPSCs, although these structures do not have self-renewing capabilities [75]. Despite the advancements, iPSC-derived organoids pose concerns regarding genomic instability, epigenetic abnormalities, and potential tumorigenicity and immunogenicity [76]. To negate this concern, organoids can be derived from embryonic or adult tissue-resident stem cells [77].

#### 4.1.2. Primary Cell-Derived Organoids

Primary human liver cells have served as valuable in vitro models for liver diseases in general due to their ability to closely represent donor tissue by retaining many molecular and cellular characteristics of their in vivo counterparts [78]. However, they face significant limitations. Specifically, they lack the self-renewal capacity and yield a limited number of cells from a single liver. This scarcity poses substantial challenges for large-scale applications and leads to high costs. Additionally, molecular and functional variations among hepatocytes from different individuals can affect the reproducibility of results [79]. Organoids generated from primary human hepatocytes have been utilized to study metabolic disorders in vitro, including modeling MASLD. In a study using organoids, MASLD was induced by inhibiting miR-122 in primary human hepatocytes using lentiviral transfection [80]. These organoids included Kupffer cells, hepatic stellate cells (HSCs), and liver sinusoidal endothelial cells. They demonstrated typical features such as inflammation, necrosis, steatosis, fibrosis, and dysregulated insulin signaling in steatosis [80]. Critical factors for the viability and survival of organoids generated from primary liver cells were found to be the liver ischemic time during operation, and the temperature variations between in vivo and in vitro conditions [81].

### 4.2. Self-Organizing Liver Organoid Models

The formation of organoids relies on cell-cell interactions among the different cell types used, resulting in less controlled outcomes and higher heterogeneity among replicates. Strategies for generating liver organoid models generally fall into two categories: self-organizing liver organoid models and bioengineered liver organoid models.

A commonly utilized method for developing human organoid models is the stepwise differentiation of PSCs into specific lineages. This technique leverages the inherent self-organizing ability of progenitor and differentiated cells to establish essential interactions, resulting in structures that closely resemble human tissue. The ability to produce multiple cell types from a single population underscores the effectiveness of using PSCs. One significant benefit of this approach is the relatively short time required to generate liver organoids (around 20 days) [79].

Multi-cellular liver models can also be created by co-culturing various cell types. PSC-derived liver organoids primarily consist of cells with fatal characteristics, which may not fully mimic the functions of mature adult cells [82]. The co-culturing method allows for the use of functionally mature cell types, addressing this limitation. Additionally, co-culturing offers precise control over the proportions of different cell types within the 3D cell model, thereby reducing batch-to-batch heterogeneity and enhancing the suitability for drug testing and screening [65]. However, direct co-culture of different liver cell types in suspension or matrices often fails to produce structures that accurately resemble liver tissue. In most co-culture methods, cells are evenly mixed and randomly distributed within the 3D model [83]. In contrast, the PSC differentiation process to generate organoids replicates morphogenesis events that occur during embryonic development, where cells form interactions and structures in response to environmental cues [84].

### 4.3. Bioengineered Liver Organoid Models

Bioengineered liver organoid models aim to improve the control over cellular interactions and orientations, facilitating the creation of larger and more complex structures with enhanced reproducibility [79]. These models hold significant promise for advancing liver disease research and therapeutic development by providing more accurate and functional representations of liver tissue. A primary challenge in developing cellular models for MASLD is the integration of a functional vascular system. This system is essential for accurately modeling various MASLD features, such as immune cell migration in the liver sinusoid during inflammation and fibrogenesis [85]. Bioengineering techniques are often utilized to recreate such vascular systems, ensuring the proper nutrient and oxygen supply necessary for sustaining cellular functions [86]. Achieving precise cell placement within multicellular models is another critical aspect of bioengineered liver organoids. Microfluidics, bioprinting, and organ scaffolds are among the key techniques employed [79]. The microfluidics platform is extensively used in organ-on-a-chip systems, which strive to recreate human tissue physiological models. These systems can range from simple single-cell-type cultures to complex multiple-cell-type co-cultures in interconnected chambers [87]. The flow system in these platforms replicates the relevant fluid shear stress observed in tissues, enabling constant exchange of fluids and biomolecules. Additionally, microfluidic systems can simulate mechanical stretch and compression observed in tissues by employing various chip designs and materials, including cell culture matrices [88]. Multiple groups have utilized the microfluidics platform to create MASLD models harboring hepatic cells or co-cultured with non-parenchymal cells (NPCs) [89]. However, the relatively low throughput, high cost, and limited materials for downstream molecular studies compared to other cell culture methods have restricted the applications of this system [79].

An alternative approach to generating organ-like cultures in vitro is the use of decellularized organ scaffolds. Human or animal organs and tissues are enzymatically decellularized, leaving behind a scaffold composed largely of the ECM. Primary or immortalized human cells are introduced into the scaffolds, and the existing ECM supports cell adhesion and repopulation [90]. This method leverages the endogenous architecture of the tissue to recreate organ-like 3D cultures with cellular distribution and interactions mimicking in vivo tissues. De l’Hortet et al. generated a large, centimeter-sized MASH model using this approach [91]. On the other hand, bioprinting methods allow for the precise placement of different cell populations within organoids to mimic liver lobule architecture. Norona et al. developed a bioprinted human liver organoid containing a compartment of primary human hepatocytes (PHHs) adjacent to a non-parenchymal cell compartment with HSCs and endothelial cells. These organoids, housed in a 24-well transwell format, showed high viability, albumin secretion, and CYP3A4 activity for 28 days. They were also more sensitive to the toxicity of trovafloxacin after 7 days of treatment compared to conventional monolayers [92].

A significant advantage of bioengineering approaches in generating human liver models is the precise control over system parameters. This control is often superior to conventional methods, and it is highly beneficial for chronic drug treatments. While bioengineered liver co-cultures may not fully recapitulate the exact architecture of the native liver, they still yield healthy and functioning liver cells. This suggests that the biochemical and biophysical microenvironment surrounding the cells is more critical for generating high-fidelity human liver models than strictly mimicking the macro-architecture of the native liver.

### 4.4. Applications of Liver Organoids in MASLD Modeling and Pathophysiology

Liver organoids offer extensive applications in biomedical research, including disease modeling, drug screening, toxicology studies, and regenerative medicine. In the context of disease modeling, they are particularly valuable for investigating monogenic diseases such as Alagille syndrome, Alpha-1 antitrypsin deficiency, Citrullinemia type 1, Cystic fibrosis, Wilson’s disease, and Wolman’s disease, as they accurately reflect the patient’s genetic background [93]. Additionally, they can recapitulate typical features of ALD. Recently, Wang and colleagues developed ESC-derived liver organoids co-cultured with fetal liver mesenchymal cells and exposed them to ethanol [94]. Besides monogenic liver diseases, they are also successfully applied to complex liver conditions, including MASLD.

In the realm of MASLD, which encompasses a spectrum of conditions with varying levels of liver damage, organoid models must be sophisticated, incorporating various liver cell types to accurately reflect cellular spatial organization [95]. Ouchi and colleagues utilized a multicellular iPSC-derived organoid model to mimic key aspects of steatosis and steatohepatitis in vitro [73]. Using 11 different healthy and diseased pluripotent stem cell lines, they developed a multi-cellular human liver organoid method composed of hepatocyte-, stellate-, and Kupffer-like cells exhibiting transcriptomic resemblance to in vivo-derived tissues. They treated liver organoids with increasing doses of FFAs, resulting in a gradual intracellular lipid accumulation. This was accompanied by the secretion of inflammatory cytokines such as TNFa, IL6, and IL8 by the Kupffer-like cells within the organoids. Prolonged FFA treatment induced hepatocyte-like cell ballooning and increased vimentin, a-smooth muscle actin expression, and collagen deposition, thus recapitulating the critical features of steatohepatitis. The optimal liver organoid system for studying MASLD should ideally involve multilineage models or co-culture systems incorporating adult tissue-derived hepatic organoids, stellate cells, and Kupffer cells [96].

Furthermore, CRISPR-Cas9 technology enhances these models by facilitating the precise introduction and correction of mutations, providing deeper insights into disease mechanisms. Recently, Deliah Hendriks and colleagues introduced FatTracer, a CRISPR-based screening platform utilizing human fetal hepatocyte organoids, to identify steatosis modulators and potential therapeutic targets [97]. This platform has successfully identified crucial genes, such as FADS2, that play a significant role in hepatic steatosis.

The disease-modeling capacity of liver organoids, derived from either adult stem cells or iPSCs, has significantly advanced drug screening and toxicology testing. One promising application is the creation of biobanks from both healthy and diseased liver cells [98]. These biobanks serve as platforms to evaluate drug efficacy against primary liver cancers (PLCs) and other liver diseases, as well as predict drug-induced liver injury (DILI), a major cause of acute liver failure. Utilizing organoids for drug discovery allows for a more accurate assessment of drug sensitivity and hepatotoxicity, thereby facilitating the identification of effective and safe therapeutic candidates [77]. In a notable study, Delilah Hendriks and colleagues developed human fetal hepatocyte organoids to model the initial stage of MASLD, specifically steatosis [97]. Steatosis was induced using three approaches: (1) loading organoids with free fatty acids to mimic excess fat accumulation, (2) introducing the PNPLA3 I148M genetic variant, which is linked to higher susceptibility to liver fat buildup, and (3) creating monogenic lipid disorders by using CRISPR to knock out the APOB and MTTP genes, both of which are critical for lipid metabolism. Through this model, they conducted drug screenings and identified compounds effective in resolving steatosis. Their research revealed that the repression of de novo lipogenesis is a common molecular pathway among these effective treatments, providing valuable insights into potential therapeutic targets for MASLD [97].

### 4.5. Limitations and Challenges of Liver Organoids

Liver organoids can revolutionize MASLD research by recapitulating key aspects of liver physiology. They offer numerous advantages over traditional models, such as maintaining long-term culture viability and enabling cryopreservation and biobanking. Additionally, liver organoids are suitable for genetic manipulation and support high-throughput screening, making them invaluable for drug discovery and toxicity testing [67]. They can be derived from minimal tissue amounts, such as needle biopsies, or from less invasive sources like iPSCs from dermal fibroblasts. Importantly, they retain the genetic background of the original tissue, preserving individual genetic variations, which makes them excellent tools for personalized medicine. By using organoids to study liver diseases and drug screening there is potential to reduce reliance on animal models. However, several challenges must be addressed to fully leverage their potential in MASLD research [99]. One significant challenge is controlling their size, shape, and cellular composition. Liver organoids usually exhibit considerable variability, affecting their reproducibility and scalability. This variability can arise from differences in the initial cell populations, culture conditions, and growth factors used. Achieving consistent and uniform organoids is crucial for their widespread implementation in research and therapeutic applications [100]. Advanced techniques such as microfluidics, bioprinting, and the incorporation of synthetic or decellularized scaffolds can enhance the structural and functional fidelity of organoids. These advancements could also help promote vascularization to address nutrient and oxygen supply issues that current organoid models lack [101]. Most current organoid models are derived from a single cell type, lacking the full cellular diversity found in vivo. This limitation hampers the ability to model complex pathophysiological processes of MASLD, characterized by intricate interactions among multiple cell types, including hepatocytes, stellate, Kupffer, and endothelial cells. Future organoid culture protocols should focus on integrating immune and hormonal components into liver organoid models, enabling the study of immune responses and hormonal regulation in liver diseases for a more comprehensive understanding of MASLD progression and treatment. Additionally, organoids derived from iPSCs or cancer cell lines may exhibit genetic and epigenetic abnormalities, impacting the accuracy of disease modeling and drug testing. Ensuring the genetic stability of organoids over long-term culture is essential for their reliability as research tools [102,103]. Notably, Takebe’s lab demonstrated that iPSC-derived hepatocyte organoids can mimic some aspects of MASLD in vitro, such as lipid accumulation and fibrosis, when treated with free fatty acids. However, further research is needed to determine whether these organoids can accurately model the progression from MASLD to MASH and subsequently to cirrhosis and HCC. Additionally, investigating the potential of patient-derived liver organoids to identify biomarkers for disease progression and develop patient-specific therapies is crucial.

## 5. Concluding Remarks and Future Perspectives of Ex Vivo Models in MASLD Research

Ex vivo models play a crucial role in elucidating the pathophysiology of MASLD, allowing for the investigation of disease mechanisms in controlled environments [12]. This review underscores the rapid advancements in MASLD research, highlighting innovative ex vivo modeling techniques that have the potential to bridge laboratory discoveries and clinical applications. Models such as PCLS and LoC systems and liver organoids provide insights into lipid metabolism, insulin resistance, inflammation, and fibrosis—key components of MASLD [79]. The majority of these models mimic the 3D architecture and cellular diversity of the liver, offering a closer representation of the in vivo environment compared to traditional 2D cultures, enabling the study of complex interactions between different cell types and the ECM. This contributes to a deeper understanding of MASLD progression and reveals critical pathways involved in disease development, such as the role of hepatic stellate cells in fibrosis and the impact of lipid accumulation on hepatocyte function [104].

Despite their potential, ex vivo models face several technical challenges and translational barriers (see also Table 1). One major challenge is maintaining liver-specific functions and phenotypes over extended time periods in culture. Primary hepatocytes, for instance, tend to lose their functionality rapidly, limiting their long-term utility [105]. Additionally, replicating the complex liver microenvironment, including the interactions between hepatocytes, stellate cells, and immune cells, remains challenging. Another significant barrier is the scalability and reproducibility of ex vivo models, which are crucial for high-throughput drug screening and large-scale studies (see also Table 1). Furthermore, translating findings from ex vivo models to clinical settings requires careful consideration of the differences between the model systems and human physiology [106]. Addressing these challenges is essential to enhance the reliability and applicability of ex vivo models in MASLD research. Advances in tissue engineering, including the development of more sophisticated scaffolds and bioreactors, are needed to overcome these limitations and improve the fidelity of ex vivo models.

The pharmaceutical industry heavily relies on effective models for drug screening and development and ex vivo tools are particularly valuable for evaluating the efficacy and toxicity of potential therapeutics for MASLD [16,107]. For instance, as already highlighted above, liver organoids allow for high-throughput screening of drug candidates, providing early insights into their impact on liver cells which in turn can be employed to test compounds in a more physiologically relevant context, improving thus the prediction of in vivo drug responses. However, controlling their size, shape, and cellular composition remains a significant challenge due to variability arising from initial cell populations, culture conditions, and growth factors (see also Table 1) [77,108]. Achieving consistent and uniform organoids is crucial for their widespread application in research and therapeutic contexts. Advanced techniques such as microfluidics, bioprinting, and the incorporation of synthetic or decellularized scaffolds can enhance the structural and functional fidelity of organoids and promote vascularization to address nutrient and oxygen supply issues [109].

The use of ex vivo models accelerates the drug discovery process and reduces the reliance on animal models, which can be time-consuming and ethically contentious [110]. These models have been instrumental in screening compounds that target key pathways in MASLD, such as lipid metabolism modulators and anti-inflammatory agents. As mentioned above, ex vivo models also facilitate the investigation of DILI by providing a platform to evaluate the hepatotoxicity of new drugs [110,111]. This contributes to the development of more accurate and non-invasive diagnostic tools for MASLD. Additionally, they can be used to study liver cell responses to various stimuli, helping identify biomarkers indicative of disease progression or therapeutic response, thereby contributing to the development of more accurate and non-invasive diagnostic tools for MASLD [112]. The identification of specific biomarkers—such as cytokines, lipid species, and genetic signatures—enhances clinical management by allowing earlier detection and better monitoring of disease progression and treatment efficacy [113].

Furthermore, ex vivo models are instrumental in advancing personalized medicine for MASLD management [114]. Patient-derived liver cells, including hepatocytes and iPSC-derived hepatocytes, can create personalized ex vivo models that reflect individual genetic and phenotypic characteristics [115]. This approach enables the assessment of tailored therapeutic strategies. The integration of ex vivo models with genomic and proteomic data facilitates comprehensive personalized medicine approaches, addressing the specific needs of MASLD patients [110,116].

Importantly, this review highlights the rapidly evolving landscape of MASLD research, emphasizing the need for innovative approaches to understanding the complexities of the disease. By detailing the advancements in ex vivo modeling techniques, this manuscript contributes to the ongoing dialogue in the field and underscores the significance of these models in bridging the gap between laboratory findings and clinical applications. The insights provided here are not only timely but also critical in guiding future research directions, particularly in the exploration of novel therapeutic targets and the development of personalized treatment strategies. In conclusion, ex vivo models offer valuable tools for advancing MASLD research, providing insights into disease mechanisms, facilitating drug discovery, aiding biomarker identification, and supporting personalized medicine. However, their application is not without challenges. Overcoming technical hurdles and translational barriers is essential to fully harness the potential of ex vivo models in understanding and treating MASLD. Continued advancements in ex vivo model technology and their integration with other research approaches will contribute significantly to the fight against MASLD. The collaboration among researchers, clinicians, and industry is crucial to translating these findings into clinical practice and ultimately improving the lives of patients with MASLD.

## Figures and Tables

**Table 1:** Comparison of Features between LoC, PCLS, and Organoids Systems.

Feature	Liver-on-a-Chip (LoC)	Precision-Cut Liver Slice (PCLS)	Liver Organoids	References
Structure	Microfluidic platforms with engineered liver tissues	Thin slices of liver tissue	3D structures derived from stem cells or primary cells	[66,67,68,69]
Cellular Composition	Hepatocytes, endothelial cells, stellate cells	Retains native liver architecture and cell diversity	Hepatocyte-like cells, cholangiocytes, stellate cells	[64,66,73]
Mimicking In Vivo Environment	Dynamic fluid flow simulating blood circulation	Maintains cell–cell and cell–matrix interactions	3D environment with cell–cell and cell–ECM interactions	[68,69,70,81]
Scalability	Moderate, dependent on microfabrication techniques	Limited by size of initial tissue slice	High, scalable to large numbers	[80,92]
Duration of Viability	Days to weeks	Hours to days	Weeks to months	[68,69,93]
Cost	Moderate, but depends on tissue source	Moderate, but depends on tissue source	Variable, depending on source and differentiation protocol	[80]
Reproducibility	High with standardized protocols	Thin slices of liver tissue	High with standardized differentiation protocols	[67,69,79]
